# Native T1 mapping and extracellular volume fraction for differentiation of myocardial diseases from normal CMR controls in routine clinical practice

**DOI:** 10.1186/s12872-021-02086-3

**Published:** 2021-06-03

**Authors:** Rawiwan Thongsongsang, Thammarak Songsangjinda, Prajak Tanapibunpon, Rungroj Krittayaphong

**Affiliations:** 1 Division of Cardiology, Department of Medicine, Faculty of Medicine Siriraj Hospital, Mahidol University, 2 Wanglang Road, Bangkoknoi, Bangkok, 10700 Thailand; 2 Her Majesty Cardiac Center, Faculty of Medicine Siriraj Hospital, Mahidol University, Bangkok, Thailand

**Keywords:** T1 mapping, Extracellular volume fraction, myocardial disease, cardiomyopathy

## Abstract

**Background:**

This study aimed to determine native T1 and extracellular volume fraction (ECV) in distinct types of myocardial disease, including amyloidosis, dilated cardiomyopathy (DCM), hypertrophic cardiomyopathy (HCM), myocarditis and coronary artery disease (CAD), compared to controls.

**Methods:**

We retrospectively enrolled patients with distinct types of myocardial disease, CAD patients, and control group (no known heart disease and negative CMR study) who underwent 3.0 Tesla CMR with routine T1 mapping. The region of interest (ROI) was drawn in the myocardium of the mid left ventricular (LV) short axis slice and at the interventricular septum of mid LV slice. ECV was calculated by actual hematocrit (Hct) and synthetic Hct. T1 mapping and ECV was compared between myocardial disease and controls, and between CAD and controls. Diagnostic yield and cut-off values were assessed.

**Results:**

A total of 1188 patients were enrolled. The average T1 values in the control group were 1304 ± 42 ms at septum, and 1294 ± 37 ms at mid LV slice. The average T1 values in patients with myocardial disease and CAD were significantly higher than in controls (1441 ± 72, 1349 ± 59, 1345 ± 59, 1355 ± 56, and 1328 ± 54 ms for septum of amyloidosis, DCM, HCM, myocarditis, and CAD). Native T1 of the mid LV level and ECV at septum and mid LV with actual and synthetic Hct of patients with myocardial disease or CAD were significantly higher than in controls.

**Conclusions:**

Although native T1 and ECV of patients with cardiomyopathy and CAD were significantly higher than controls, the values overlapped. The greatest clinical utilization was found for the amyloidosis group.

**Supplementary Information:**

The online version contains supplementary material available at 10.1186/s12872-021-02086-3.

## Introduction

Cardiac magnetic resonance (CMR) has been increasingly used for the assessment of patients with suspected or known heart disease. Late gadolinium enhancement (LGE) can illustrate myocardial fibrosis. The common type of fibrosis detected by LGE is replacement fibrosis which happened after myocardial injury [1]. T1 mapping can be performed to demonstrate myocardial tissue characterization. It can detect and quantify infiltrative diseases and diffuse fibrosis [2]. The Society for Cardiovascular Magnetic Resonance and the CMR Working Group of the European Society of Cardiology (ESC) recommended the integration of T1 mapping into routine CMR practice as a key tissue characterization [3]. Myocardial T1 is prolonged in fibrosis and edema, but reduced in lipid accumulation [4]. The extracellular volume fraction (ECV), which represents percentages of extracellular space of the myocardial tissue, can be derived from pre- and post-contrast T1 values of myocardium and blood pool in combination with hematocrit (Hct) using the established formula [5]. An increased ECV value indicates the presence of excessive collagen deposition or fibrosis, such as in amyloidosis or myocardial infarction [6]. When the actual Hct is unavailable, a synthetic Hct value can be derived from the relationship between Hct and the longitudinal relaxation rate of blood [7]. The ECV is associated with cardiovascular outcomes [8–10]. ECV associated with the all-cause mortality and composite HF endpoints in non-ischemic cardiomyopathy (NICM) patients, using values derived from septal T1 measurements [8], and associated with composite endpoints of hospitalization for heart failure and cardiac death in HFpEF population, using T1 values derived from the whole segment of LV [9]. Meta-analysis of six studies reported that cardiovascular disease patients including NICM, amyloidosis, and small amount of CAD, who have an increase in ECV value had a significantly higher incidence of cardiovascular death and combined cardiac events [10]. The results from previous studies were based on a relatively small sample size and showed inconsistent findings in patients with CAD [11–13].

The primary objective of this study was to determine T1 values and ECV in distinct types of myocardial disease, including amyloidosis, dilated cardiomyopathy (DCM), hypertrophic cardiomyopathy (HCM), myocarditis, and coronary artery disease (CAD) compared to controls. The secondary objective was to determine the diagnostic yield and cut-off values of T1 and ECV for differentiation of myocardial disease and CAD compared to controls.

## Methods

### Patient population

This study was approved by the Siriraj Institutional Review Board (SIRB) of the Faculty of Medicine Siriraj Hospital, Mahidol University, Bangkok, Thailand. According to committee of SIRB, consent to participate was not required due to the retrospective nature of this study. The study was conducted in compliance with the principles of the Declaration of Helsinki, International Conference on Harmonization Good Clinical Practice guidelines on non-interventional studies. Patients who underwent clinical stress/viability protocol of CMR 3 Tesla with routine T1 mapping at Siriraj Hospital during July 2017 to December 2019 for clinical indication were enrolled. Subjects were categorized based on CMR findings into the myocardial disease group, the CAD group, or the control group. The myocardial disease group included patients diagnosed with amyloidosis, DCM, HCM, or myocarditis.

We used established criteria in combination with CMR criteria for the diagnosis of amyloidosis [14], DCM [15], HCM [16], acute and convalescent myocarditis [17, 18], and CAD [19]. Due to the retrospective nature of this study, patients who had generally accepted contraindications to CMR were not included. Additional exclusion criteria included: (1) artifact of partial volume that was uncorrectable by motion correction program that cause contaminated T1 mapping; (2) moderate or severe valvular heart disease; (3) pericardial disease, congenital heart disease, and other specific cardiomyopathies, such as arrhythmogenic right ventricular cardiomyopathy, restrictive cardiomyopathy, hypertensive cardiomyopathy, stress induced cardiomyopathy, eosinophilic cardiomyopathy, hemochromatosis, and endomyocardial fibrosis; and, (4) patients with acute myocardial infarction.

**Fig. 1:**
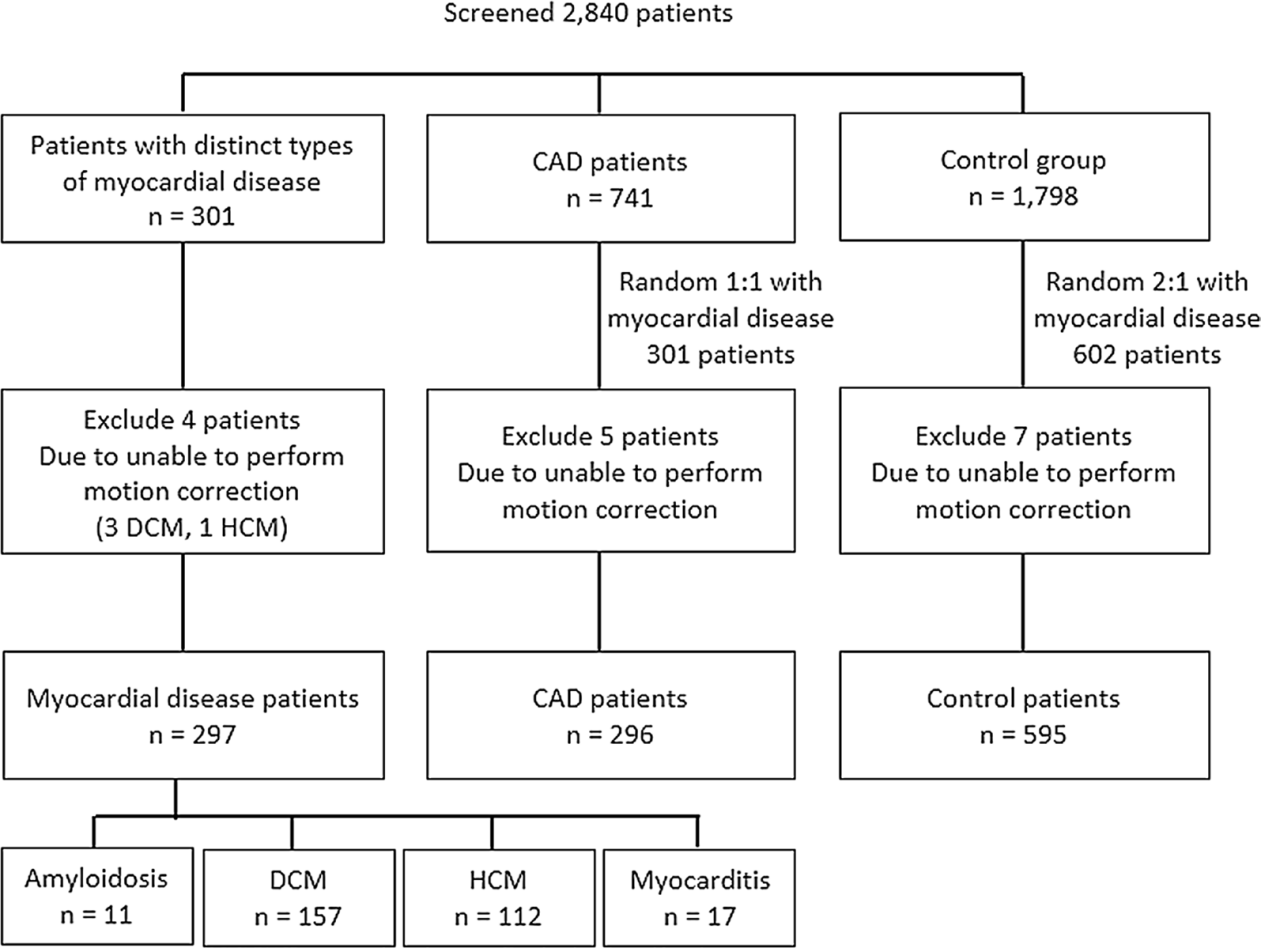
Details of the study population. *CAD* coronary artery disease, *DCM* dilated cardiomyopathy, *HCM* hypertrophic cardiomyopathy

Figure 1 demonstrates the patient enrollment and group assignment protocol. CAD patients were enrolled at a 1:1 ratio with myocardial disease patients (n = 301 in each group). The control group comprised patients who did not meet the diagnostic criteria for either myocardial disease or CAD, had no known heart disease, and normal CMR study. The number of patients in the control group was 2 times that of both of the other 2 groups (n = 602).

### Cardiac magnetic resonance (CMR) protocol

MR imaging examinations were performed using a commercial Ingenia 3.0T MR system (Philips Medical Systems, Best, the Netherlands). The routine CMR protocol included black blood axial plain images, steady-state free precession (SSFP) cine images of standard long-axis, 2-chamber, 3-chamber, 4-chamber views, and native modified Look-Locker inversion recovery (MOLLI) T1 mapping. Native T1 mapping was performed in mid-diastole with breath-holding technique and using MOLLI in a single mid-ventricular short axis slice (TR 2.2 ms, TE 1.8 ms, 8 different TIs, matrix 152 × 150, field of view 300 × 300 mm^2^, flip angle 20°, SENSE 2, and slice thickness 10 mm) [20]. The 5-(3)-3 MOLLI sequence used in this study comprised one inversion pulse with T1 sampling performed over 5 acquisition heartbeats, followed by 3 recovery heartbeats, and a second inversion pulse followed by 3 acquisition heartbeats [20]. Ten minutes after intravenous administration of 0.15 mmol gadolinium-based MRI contrast agent [gadoterate meglumine (Dotarem®), gadobutrol (Gadovist®), and gadopentetate dimeglumine (Magnevist®)] per kg body weight, a T1-weighted Inversion Recovery Fast Low Angle Shot (3D IR-TFE) sequence was acquired in standard long and short axis views (TR 3.4 ms, TE 1.14ms, matrix 152 × 149, field of view 270 × 320 to 270 × 380 mm^2^, flip angle 15°, SENSE 2.5, and slice thickness 8 mm) to assess LGE. Post contrast T1 mapping was performed.

### CMR imaging analysis

**Fig. 2:**
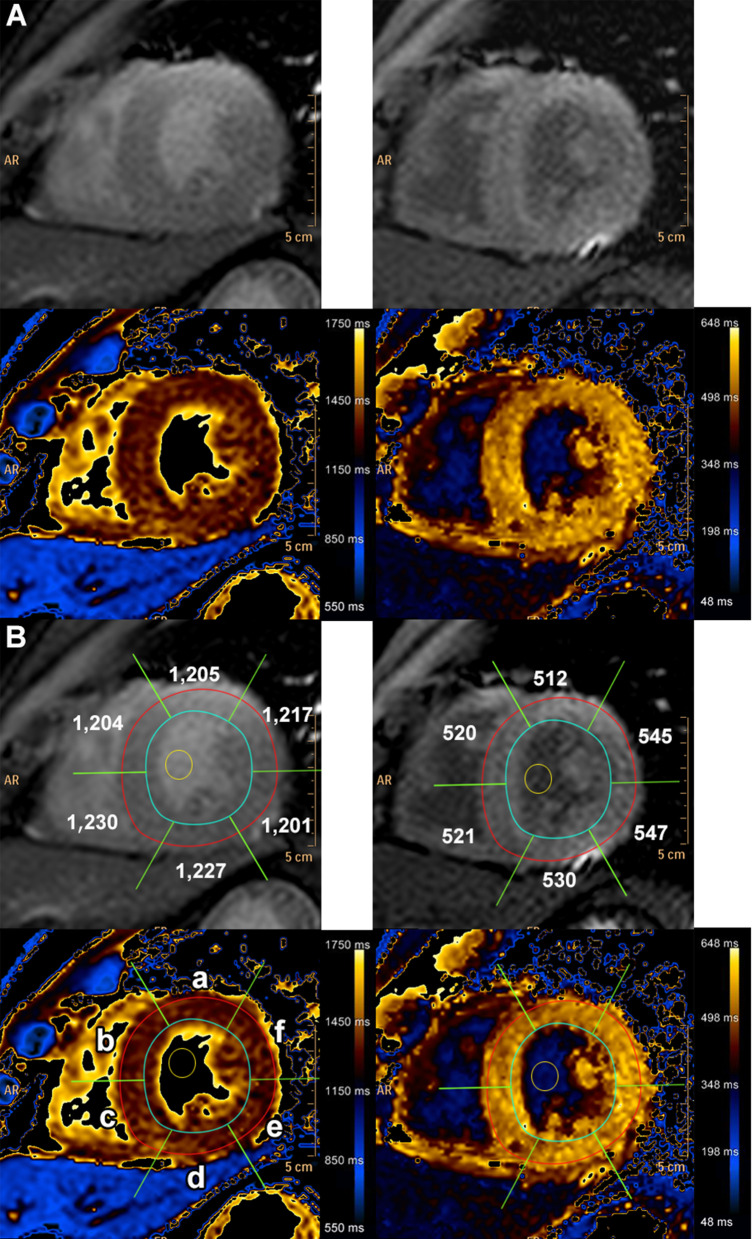
T1 mapping is displayed in color scale. **A** upper panels, native T1 and T1 enhanced; lower panels, native T1 and T1 enhanced with color scale. **B** upper panels, T1 map with color scale and region of interest. Mid-left ventricular short axis (Mid LV) native T1 mapping showed endocardial (blue line) and epicardial (red line) contours, as well as the region-of-interest for the calculation of the average myocardial T1 value. Lower panels, the subdivided 6 segments of mid-left ventricular short axis myocardium are represented, as follows: **a** mid anterior wall, **b** mid anteroseptal wall, **c** mid inferoseptal wall, **d** mid inferior wall, **e** mid inferolateral wall, and **f** mid anterolateral wall

All routine CMR analyses were performed using commercially available software (IntelliSpace Portal, version 9; Philips Healthcare, Best, the Netherlands). Routine T1 relaxation maps were also obtained using IntelliSpace Portal (version 9). The region of interest (ROI) was drawn in the myocardium of the mid left ventricular (LV) short axis slice, in the myocardium at the interventricular septum of mid LV slice and the left ventricular blood pool. Native T1 and ECV values were derived from pre- and post-contrast T1 mapping. Greyscale and color mappings were displayed with software default color scheme (Fig. 2A). The mid LV myocardium slice was selected from the region that includes the entire length of the papillary muscles [21]. The ROI was drawn with adequate margins intended to separate myocardium from the area prone to partial volume averaging, such as the area between myocardium and blood. The papillary muscles were excluded as part of the LV myocardium. For the blood pool ROI, care was taken to avoid the papillary muscles. Motion correction was applied when the source images have a different cardiac position between each slice to avoid motion effect on T1 measurement [11, 22, 23]. After the initial image affine registration step of the ROI, the source image was equally subdivided into 6 smaller ROIs to represent mid anterior wall, amid anteroseptal wall, mid inferoseptal wall, mid inferior wall, mid inferolateral wall, and mid anterolateral wall (Fig. 2B). The mid anteroseptal wall and mid inferoseptal wall represent the mid LV septum [21]. Each subdivision underwent another affine transformation to align the features of the target image ROIs with the corresponding ROIs in the source phase image. For CAD patients, the subdivision segment with ischemic-related scar was excluded from ROI of native T1 analysis. However, the subdivision segment with non-ischemic LGE scar was still included [3, 24, 25]. The ECV was calculated using the formula [5]:

$$\text{ECV}=(1-\text{hematocrit})\,\frac{\frac{1}{{\text{post contrast T1 myo}}}-\frac{1}{{\text{native T1 myo}}}}{\frac{1}{{\text{post contrast T1 blood}}}-\frac{1}{{\text{native T1 blood}}}}$$

The actual Hct value was determined either on the day of or within 6 months of the CMR study. For patients without blood sampling, ECV was calculated using a synthetic Hct value that was derived from the relationship between hematocrit and the longitudinal relaxation rate of blood according to the formula: Synthetic Hct = 869.7 × (1/T1blood)-0.071 [7]. This synthetic Hct formula was derived from pre-contrast blood T1 values acquired by MOLLI method on a 3.0 Tesla Philips system (Achieva-dStream, Philips) [7].

### Statistical analysis

Statistical analysis was performed using SPSS Statistics version 18 (SPSS, Inc., Chicago, IL, USA). Normally distributed data are reported as mean plus/minus standard deviation (SD), and categorical data are given as number and percentage. Differences in the average T1 and ECV values between control group and diseased groups were evaluated using independent samples *t*-test. For comparison of two and more than two normally distributed variables, Student’s *t*-test and one-way analysis of variance (ANOVA) was used, respectively. Sensitivity, specificity, diagnostic accuracy, positive predictive value (PPV), negative predictive value (NPV), area under the curve (AUC), and cutoff values of native T1 and ECV values to distinguish between controls and the 5 evaluated heart diseases were calculated using receiver operating characteristic (ROC) curve analysis. Comparison of diagnostic yield for detecting diseased cases was performed by the McNemar’s test. Comparison of native T1 and ECV between 2 different regions of interest was performed using Pearson’s correlation coefficient. Bland-Altman plot, which was used to determine the level of test-retest reliability (calculated as percentage differences from the mean of 1.96 SDs), was performed using MedCalc statistical software program (MedCalc Software Ltd, Ostend, Belgium). Statistical significance was defined as a *p*-value less than 0.05.

## Results

We enrolled 2840 patients who underwent routine CMR T1 mapping protocol (Fig. 1). Patients in CAD group were randomly selected to match the number of patients in myocardial disease group. Patients in the control group were also randomized to have the sample size twice the size of the myocardial disease group. The result of that enrollment phase yielded 301 patients in the myocardial disease group, 301 patients in the CAD group, and 602 patients in the control group for a total of 1,204 patients. Sixteen additional patients (4 myocardial disease, 5 CAD, and 7 controls) had to be excluded due to uncorrectable artifacts caused by breathing and/or arrhythmia. A final study population size of 1188 patients were analyzed.

The mean age of patients was 66.7 ± 13.7 years, and 48.0% were males. Patient baseline demographic and clinical characteristics are shown in Table 1. Of the 296 CAD patients, 109 patients had no scar, and 187 patients had scarring on LGE. Three patients had scars in all segments of mid LV myocardium and was all excluded from ROI drawing. Thus, mid LV native T1 value of them could not be derived. There were 49 patients without septum native T1 value also due to exclusion of scar area in both segments of septum from ROI.

**Table 1:** Baseline demographic and clinical characteristics

Characteristics	All (N = 1188)	Amyloidosis (n = 11)	DCM (n = 157)	HCM (n = 112)	Myocarditis (n = 17)	CAD (n = 296)	Control (n = 595)
Male gender	570 (48.0%)	6 (54.5%)	89 (56.7%)	59 (52.7%)	14 (82.4%)	198 (66.9%)	204 (34.3%)
Age, yrs	66.7 ± 13.7	57.2 ± 13.7	59.2 ± 16.6	64.2 ± 15.9	54.7 ± 23.0	71.3 ± 11.0	67.4 ± 12.0
Weight, kg	65.1 ± 14.7	61.1 ± 18.9	64.7 ± 18.9	64.0 ± 13.1	65.8 ± 19.2	64.8 ± 12.8	65.6 ± 14.3
Height, cm	159.8 ± 12.6	163.5 ± 11.5	162.0 ± 13.2	160.1 ± 10.8	164.7 ± 8.6	161.0 ± 12.5	158.4 ± 12.7
BMI, ml/m^2^	25.2 ± 4.8	22.4 ± 4.3	24.3 ± 5.4	24.9 ± 4.3	23.9 ± 5.4	24.8 ± 4.4	25.9 ± 4.9
Hematocrit, %	39.1 ± 5.3	38.8 ± 3.2	38.9 ± 6.9	40.7 ± 5.0	40.6 ± 7.3	38.4 ± 5.6	39.1 ± 4.7
eGFR, ml/min/1.73m^2^	70.2 ± 24.6	88.8 ± 30.1	71.6 ± 29.7	66.7 ± 24.01	78.2 ± 29.9	64.1 ± 23.9	74.3 ± 21.9
Dyslipidemia	704 (59.3%)	3 (27.3%)	58 (36.9%)	58 (51.8%)	5 (29.4%)	206 (69.6%)	374 (62.9 %)
Diabetes mellitus	396 (33.3%)	1 (9.1%)	37 (23.6%)	28 (25.0%)	4 (23.5%)	137 (46.3%)	189 (31.8%)
Hypertension	786 (66.2%)	4 (36.4%)	83 (52.9%)	67 (59.8%)	6 (35.3%)	231 (98.0%)	395 (66.4%)
Heart failure	161 (13.6%)	6 (54.5%)	52 (33.1%)	13 (11.6%)	3 (17.6%)	50 (16.9%)	37 (6.2%)

Data are presented as number and percentage or mean ± standard deviation

*BMI* body mass index, *eGFR* estimated glomerular filtration rate, *DCM* dilated cardiomyopathy, *HCM* hypertrophic cardiomyopathy, *CAD* coronary artery disease

### Comparisons of native T1 and ECV for discriminating between myocardial disease and controls, and between CAD and controls

**Fig. 3:**
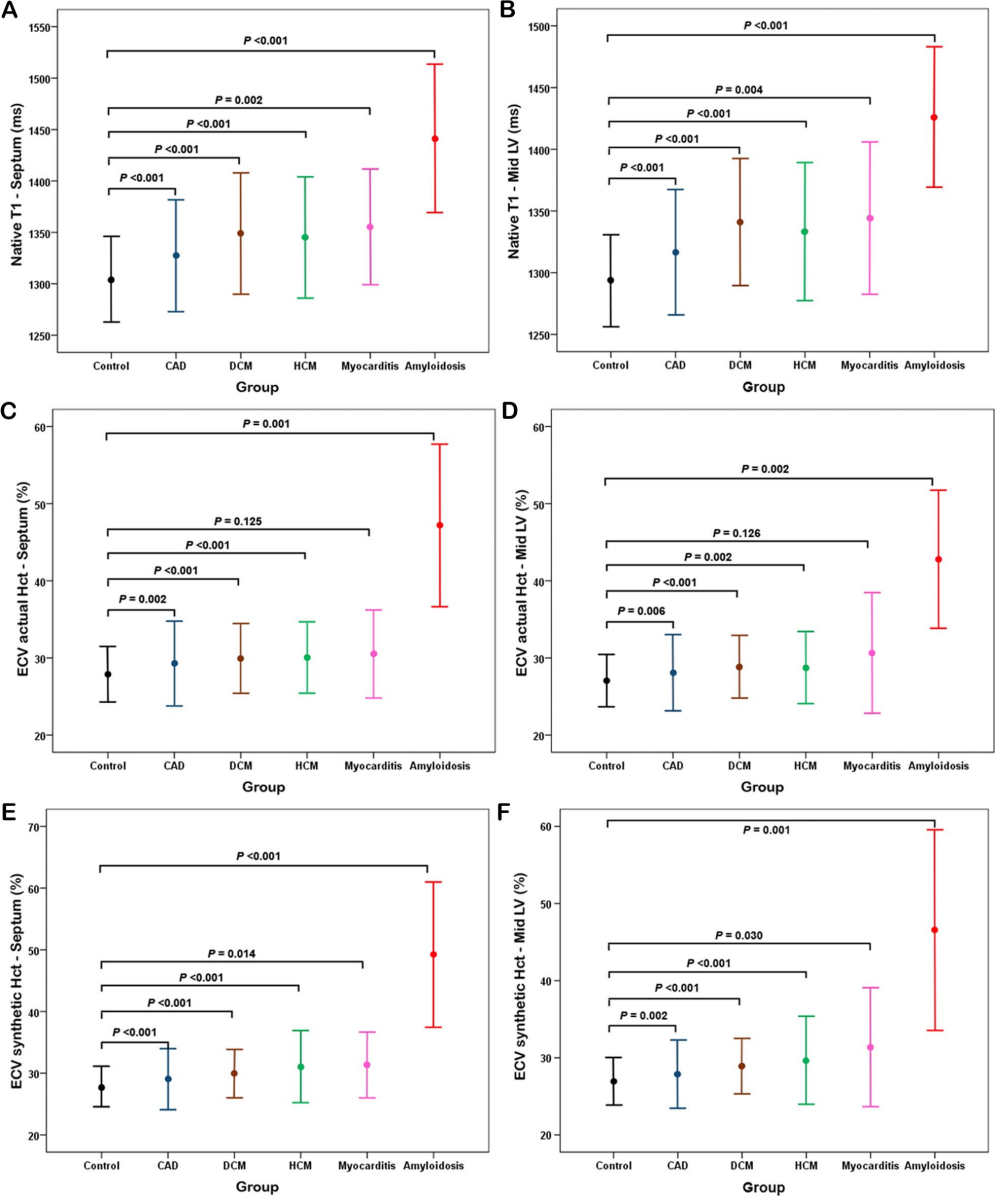
Comparison of native T1 values and ECV among myocardial diseases, coronary artery disease (CAD) and controls (DCM, dilated cardiomyopathy; HCM, hypertrophic cardiomyopathy). **A** native T1 at septum, **B** native T1 at mid LV, **C** ECV using actual Hct at septum, **D** ECV using actual Hct at mid LV, **E** ECV using synthetic Hct at septum, **F** ECV using synthetic Hct at mid LV

The average native T1 value and ECV are presented in Tables 2 and 3, respectively. Statistical analysis showed significant difference between the control group and the myocardial disease group for all mid LV native T1 values (*p* < 0.001), and for all septum native T1 values (*p* < 0.001). The native T1 values and ECV between myocardial disease and CAD and controls are shown in Fig. 3. Although native T1 and ECV were significantly higher in cardiomyopathies and CAD compared to controls, there were significant overlaps between cardiomyopathies, CAD and controls except for amyloidosis. The results of ROC curve analysis with corresponding cutoff values of native T1 for the differentiation of each myocardial disease subgroup and CAD from controls are presented in Table 4.

**Table 2:** Comparison of native T1 values between patients with myocardial disease or coronary artery disease and control group

Group	Mid LV short-axis native T 1 (ms)			Group	Septum native T1 (ms)		
	Mean ± SD	95% CI	p-value		Mean ± SD	95% CI	p-value
Amyloidosis (n = 11)	1426 ± 57	1392–1460	***< 0.001***	Amyloidosis (n = 11)	1441 ± 72	1393–1490	***< 0.001***
DCM (n = 157)	1341 ± 52	1333–1349	***< 0.001***	DCM (n = 157)	1349 ± 59	1340–1358	***< 0.001***
HCM (n = 112)	1333 ± 56	1323–1344	***< 0.001***	HCM (n = 112)	1345 ± 59	1334–1356	***< 0.001***
Myocarditis (n = 17)	1344 ± 62	1315–1374	***0.004***	Myocarditis (n = 17)	1355 ± 56	1327–1384	***0.002***
CAD (n = 293)	1316 ± 51	1310–1322	***< 0.001***	CAD (n = 247)	1328 ± 54	1321–1334	***< 0.001***
Control (n = 595)	1294 ± 37	1291–1297		Control (n = 638)	1304 ± 42	1301–1308	

A *p*-value < 0.05 indicates statistical significance

*SD* standard deviation, *CI* confidence interval, *DCM* dilated cardiomyopathy, *HCM* hypertrophic cardiomyopathy, *CAD* coronary artery disease

**Table 3:** Comparison of ECV using actual and synthetic Hct between myocardial disease or coronary artery disease and control group

Group	Mid LV short-axis ECV with actual Hct (%)			Septum ECV with actual Hct (%)				Mid LV short-axis ECV with synthetic Hct (%)				Septum ECV with synthetic Hct (%)			
n	Mean ± SD	95% CI	p	n	Mean ± SD	95% CI	p	n	Mean ± SD	95% CI	P	n	Mean ± SD	95% CI	p
Amyloidosis (n = 8)	42.8 ± 9.0	35.3–50.3	***0.002***	(n = 8)	47.2 ± 10.5	38.4–56.0	***0.001***	(n = 11)	46.6 ± 13.0	38.9–54.3	***0.001***	(n = 11)	49.3 ± 11.8	41.4–57.2	***< 0.001***
DCM (n = 116)	28.9 ± 4.1	28.1–29.6	***< 0.001***	(n = 116)	29.9 ± 4.5	29.1–30.8	***< 0.001***	(n = 157)	28.9 ± 3.6	28.4–29.5	***< 0.001***	(n = 157)	30.0 ± 3.9	29.4–30.6	***< 0.001***
HCM (n = 84)	28.7 ± 4.6	27.7–29.7	***0.002***	(n = 84)	30.1 ± 4.6	29.1–31.1	***< 0.001***	(n = 112)	29.7 ± 5.7	28.6–30.7	***< 0.001***	(n = 112)	31.1 ± 5.8	30.0-32.2	***< 0.001***
Myocarditis (n = 13)	30.6 ± 7.8	25.9–35.4	0.126	(n = 13)	30.5 ± 5.7	27.1–34.0	0.125	(n = 17)	31.4 ± 7.7	27.7–35.1	***0.030***	(n = 17)	31.3 ± 5.3	28.6–34.1	***0.014***
CAD (n = 223)	28.1 ± 5.0	27.5–28.7	***0.006***	(n = 186)	29.3 ± 5.5	28.5–30.1	***0.002***	(n = 293)	27.8 ± 4.3	27.3–28.3	***0.002***	(n = 247)	29.1 ± 4.9	28.5–29.7	***< 0.001***
Control (n = 465)	27.1 ± 3.4	26.8–27.4	–	(n = 465)	27.9 ± 3.6	27.6–28.3	–	(n = 595)	26.9 ± 3.1	26.7–27.2	–	(n = 595)	27.0 ± 3.3	27.5–28.0	–

A *p*-value < 0.05 indicates statistical significance

*ECV* extracellular volume fraction, *Hct* hematocrit, *CAD* coronary artery disease, *SD* standard deviation, *CI* confidence interval, *DCM* dilated cardiomyopathy, *HCM* hypertrophic cardiomyopathy

**Table 4:** Cutoff values from receiver operating characteristic (ROC) curve analysis of native T1 in myocardial disease and CAD

	Cutoff (ms)	AUC	*p*-value	Specificity (%) (95% CI)	Sensitivity (%) (95% CI)	PPV (%) (95% CI)	NPV (%) (95% CI)	Diagnostic Accuracy (%) (95% CI)
*Amyloidosis (n = 11)*								
Mid LV short-axis native T1	1,341	0.990	***< 0.001***	93.9 (91.7–95.6)	100 (74.1–100.0)	23.4 (13.6–37.2)	100 (99.3–100)	94.1 (91.9–95.7)
Septum native T1	1,370	0.936	***< 0.001***	99.7 (98.8–99.9)	81.8 (52.3–94.9)	81.8 (52.3–94.9)	99.7 (98.8–99.9)	99.3 (98.3–99.7)
*DCM (n = 157)*								
Mid LV short-axis native T1	1,338	0.783	***< 0.001***	90.9 (88.3–93.3)	56.1 (48.2–63.6)	62.0 (53.8–69.5)	88.7 (85.9–91.0)	83.6 (80.8–86.1)
Septum native T1	1,341	0.736	***< 0.001***	94.3 (92.1–95.9)	50.3 (42.6–58.0)	69.9 (60.9–77.6)	87.8 (85.0–90.1)	85.1 (82.4–87.5)
*HCM (n = 112)*								
Mid LV short-axis native T1	1,333	0.723	***< 0.001***	86.9 (83.9–89.4)	53.6 (44.4–62.5)	43.5 (35.5–51.8)	90.9 (88.2–93.0)	81.6 (78.6–84.3)
Septum native T1	1,346	0.711	***< 0.001***	96.0 (94.1–97.3)	40.2 (31.6–49.4)	65.2 (53.4–75.4)	89.5 (86.9–91.6)	87.1 (84.5–89.4)
*Myocarditis (n = 17)*								
Mid LV short-axis native T1	1,355	0.710	***0.003***	98.3 (96.9–99.1)	41.2 (21.6–64.0)	41.2 (21.6–64.0)	98.3 (96.9–99.1)	96.7 (95.0–97.9)
Septum native T1	1,360	0.752	***< 0.001***	99.2 (98.0–99.6)	35.3 (17.3–58.7)	54.5 (28.0–78.7)	98.2 (96.8–99.0)	97.4 (95.8–98.4)
*CAD*								
Mid LV short-axis native T1 (n = 293)	1,342	0.621	***< 0.001***	94.3 (92.1–95.9)	30.7 (25.7–36.2)	72.6 (64.1–79.7)	73.4 (70.2–76.4)	73.3 (70.3–76.1)
Septum native T1 (n = 247)	1,343	0.618	***< 0.001***	94.6 (92.5–96.2)	28.7 (23.5–34.7)	68.9 (59.4–77.1)	76.2 (73.0–79.1)	75.3 (72.3–78.1)

A *p*-value < 0.05 indicates statistical significance

*CAD* coronary heart disease, *AUC* area under the ROC curve, *CI* confidence interval, *PPV*, positive predictive value, *NPV* negative predictive value, *DCM* dilated cardiomyopathy, *HCM* hypertrophic cardiomyopathy

**Fig. 4:**
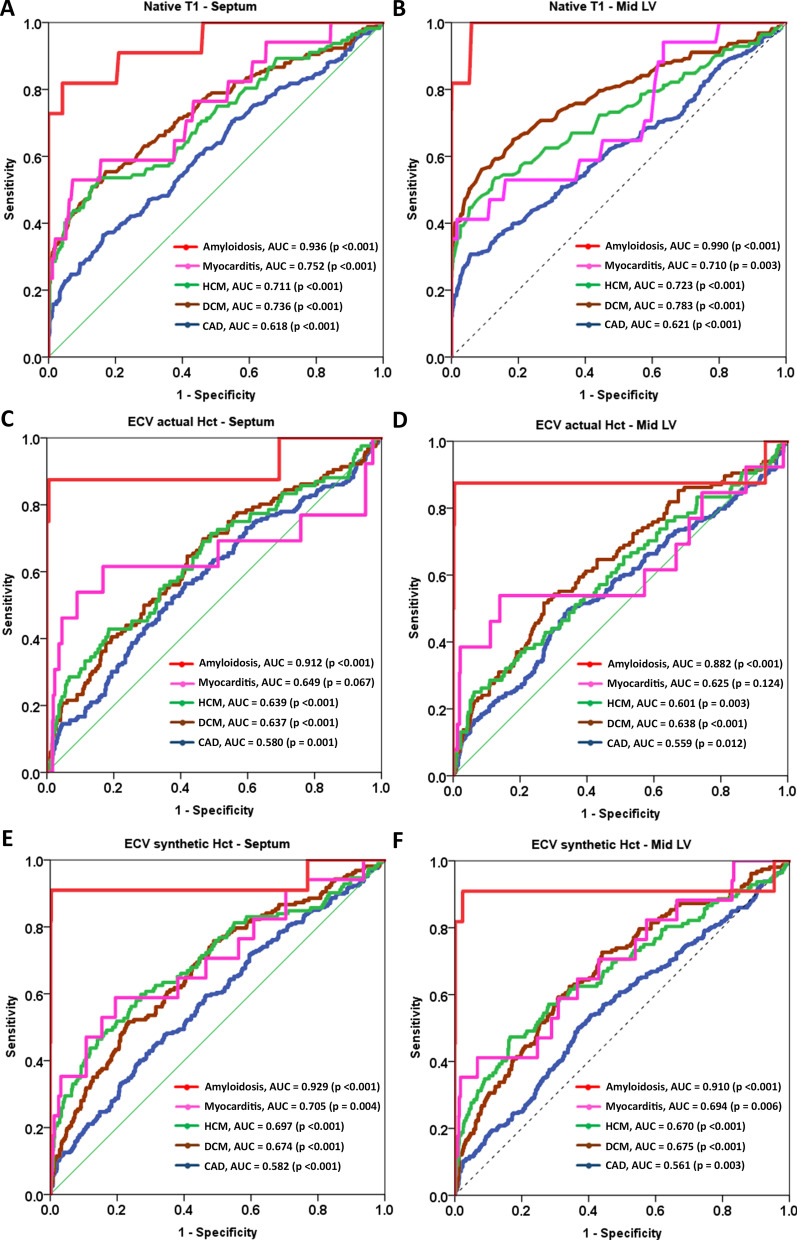
Receiver operating characteristic curve of native T1 and ECV for the diagnosis of amyloidosis, myocarditis, hypertrophic cardiomyopathy (HCM), dilated cardiomyopathy (DCM), and coronary artery disease (CAD). AUC = area under the curve. **A** Native T1–septum, **B** Native T1–mid LV, **C** ECV actual Hct–septum, **D** ECV actual Hct–mid LV, **E** ECV synthetic Hct–septum and **F** ECV synthetic Hct–mid LV

There were 909 patients who had actual Hct, and their ECV values were calculated using both actual Hct and synthetic Hct. There were 279 patients who did not have actual Hct, so their ECV values were calculated using only synthetic Hct. Statistical analysis showed significant difference between the control group and the myocardial disease group for all mid LV ECV values (*p* < 0.001) and septum ECV values (all *p* < 0.001), similar to the comparisons for native T1 value. Similar findings were demonstrated for remote area of CAD groups versus controls. The results of ROC curve analysis to evaluate for the ability of ECV to differentiate each myocardial disease subgroup and CAD from controls are presented in Table 5. Figure 4 demonstrated ROC curves of native T1 (4 A and 4B) and ECV (4 C to 4 F) for the differentiation of cardiomyopathies, CAD compared to controls. The AUCs were in the range of 0.56 to 0.99 with significant p-values. The maximum AUC was for the differentiation of cardiac amyloidosis from controls.

**Table 5:** Cutoff values from receiver operating characteristic (ROC) curve analysis of mid LV short-axis ECV in myocardial disease and CAD

	Cutoff (%)	AUC	*p*-value	Specificity (%) (95% CI)	Sensitivity (%) (95% CI)	PPV (%) (95% CI)	NPV (%) (95% CI)	Diagnostic Accuracy (%) (95% CI)
*Amyloidosis*								
Mid LV short-axis actual ECV (n = 8)	40.0	0.882	***< 0.001***	99.6 (98.4–99.9)	87.5 (52.9–97.8)	77.8 (45.3–93.7)	99.8 (98.8–100)	99.4 (98.2–99.8)
Mid LV short-axis synthetic ECV (n = 11)	32.6	0.910	***< 0.001***	97.6 (96.1–98.6)	81.8 (52.3–94.9)	39.1 (22.2–59.2)	99.7 (98.8–99.9)	97.4 (95.8–98.4)
*DCM*								
Mid LV short-axis actual ECV (n = 116)	28.8	0.638	***< 0.001***	72.9 (68.7–76.7)	51.7 (42.7–60.6)	32.3 (26.0-39.3)	85.8 (82.0-88.9)	68.7 (64.8–72.3)
Mid LV short-axis synthetic ECV (n = 157)	27.0	0.675	***< 0.001***	55.6 (51.6–59.6)	72.6 (65.2–79.0)	30.2 (25.8–35.0)	88.5 (84.9–91.4)	59.2 (55.6–62.6)
*HCM*								
Mid LV short-axis actual ECV (n = 84)	31.8	0.601	***0.003***	93.8 (91.2–95.6)	25.0 (17.0-35.2)	42.0 (29.4–55.8)	87.4 (84.2–90.0)	83.2 (79.9–86.1)
Mid LV short-axis synthetic ECV (n = 112)	29.7	0.670	***< 0.001***	83.7 (80.5–86.4)	46.4 (37.5–55.6)	34.9 (27.7–42.8)	89.2 (86.4–91.6)	77.8 (74.6–80.7)
*Myocarditis*								
Mid LV short-axis actual ECV (n = 13)	30.5	0.625	0.124	85.4 (81.9–88.3)	53.8 (29.1–76.8)	9.3 (4.6–18.0)	98.5 (96.8–99.3)	84.5 (81.0-87.5)
Mid LV short-axis synthetic ECV (n = 17)	31.3	0.694	***0.006***	92.9 (90.6–94.7)	41.2 (21.6–64.0)	14.3 (7.1–26.7)	98.2 (96.8–99.0)	91.5 (89.0-93.5)
*CAD*								
Mid LV short-axis actual ECV (n = 223)	27.9	0.559	***0.012***	65.4 (60.9–69.6)	49.3 (42.8–55.8)	40.6 (34.9–46.5)	72.9 (68.4–76.9)	60.2 (56.5–63.8)
Mid LV short-axis synthetic ECV (n = 293)	27.3	0.561	***0.003***	58.5 (54.5–62.4)	53.9 (48.2–59.5)	39.0 (34.4–43.8)	72.0 (67.9–75.9)	57.0 (53.7–60.2)

A *p*-value < 0.05 indicates statistical significance

*CAD* coronary heart disease, *AUC* area under the ROC curve, *CI* confidence interval, *PPV* positive predictive value, *NPV* negative predictive value, *ECV* extracellular volume fraction, *DCM* dilated cardiomyopathy, *HCM* hypertrophic cardiomyopathy

**Fig. 5:**
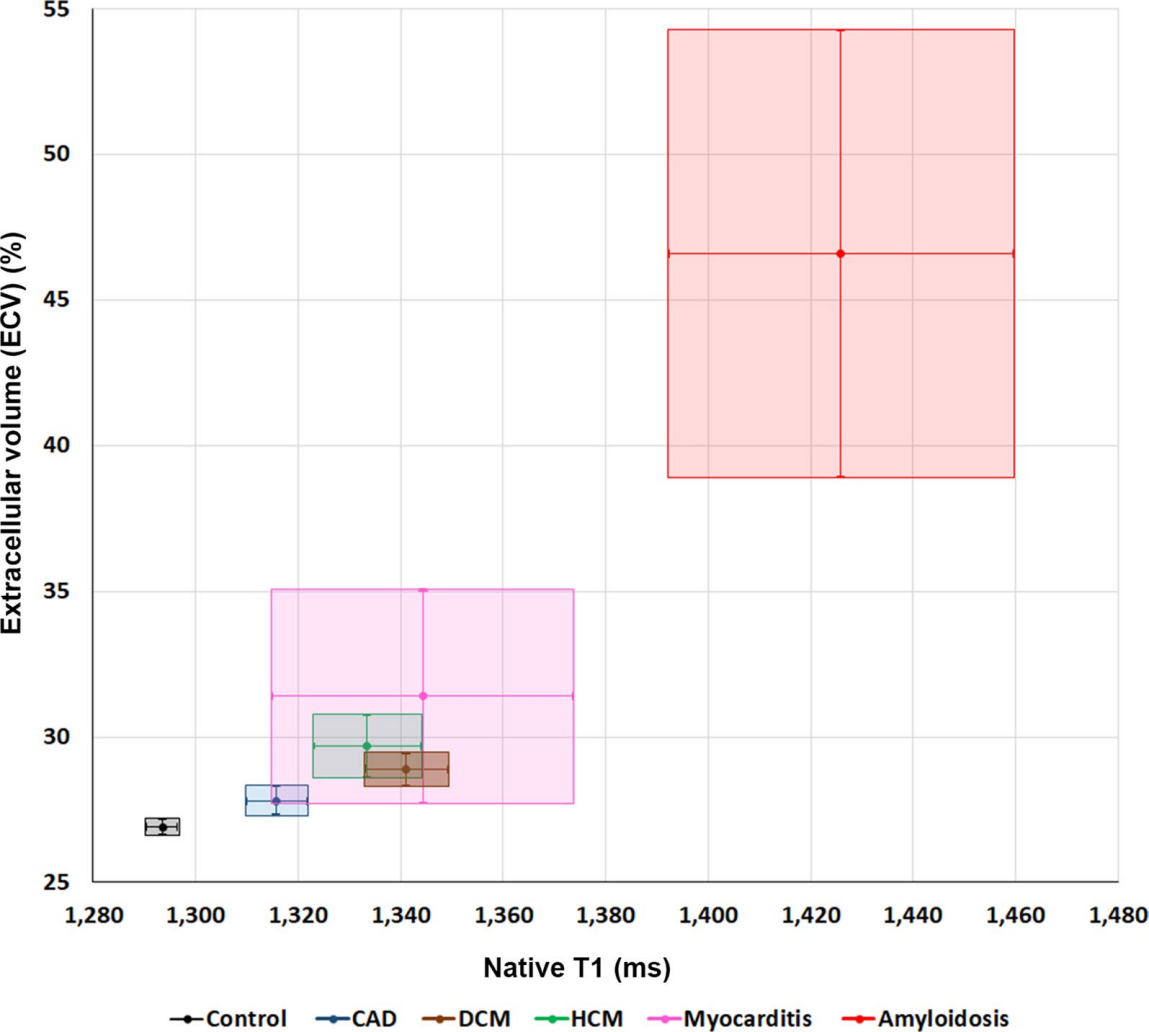
95% Confidence interval (CI) box graph showing comparison of native T1 at mid LV and extracellular volume (ECV) using synthetic Hct at mid LV in the myocardial diseases, coronary artery disease (CAD), and control groups (DCM, dilated cardiomyopathy; HCM, hypertrophic cardiomyopathy)(Horizontal dimension of each box indicates 95% CI of native T1, vertical dimension indicates 95% CI of ECV, mid-point indicates mean value of native T1 and ECV)

The relation of native T1 (at mid LV) and ECV values (at mid LV using synthetic Hct) among the different groups are demonstrated in Fig. 5. Mid LV was chosen since it might reflect diffuse disease more than septum and ECV using synthetic Hct was chosen to reflect the data of the whole group. Figure 5 which showed 95% area of each group, demonstrated that the overlapped area of each box were less with the ECV compared to native T1 which indicated that ECV would have an additional value for the differentiation of disease compared to control. To explore whether ECV data have incremental value to native T1 data alone for the diagnosis of myocardial diseases and CAD, we performed additional analysis on comparing diagnostic yield of detecting diseased cases using McNemar’s test. The results are shown in Table 6. The results showed that adding ECV to native T1 significantly increased diagnostic sensitivity of DCM and CAD but not significantly increased for amyloidosis, HCM and myocarditis.

**Table 6:** Sensitivity of native T1, ECV using synthetic hematocrit at mid LV and combination (native T1 followed by ECV if T1 was normal) in the disgnosis of amyloidosis, dilated cardiomyopathy (DCM0, hypertrophic cardiomyopathy (HCM), myocarditis, and coronary artery disease (CAD). P-values of comparison of combination of test and single test based on McNemar’s test are shown

Test		Amyloidosis (n = 11)	DCM (n = 157)	HCM (n = 112)	Myocarditis (n = 17)	CAD (n = 293)
1	Native T1	11 (100%)	88 (56.1%)	60 (53.6%)	7 (41.2%)	90 (30.7%)
2	ECV synthetic	9 (81.8%)	114 (72.6%)	52 (46.4%)	7 (41.2%)	158 (53.9%)
3	T1 and ECV synthetic	11 (100.0%)	125 (79.6%)	60 (53.6%)	7 (41.2%)	175 (59.7%)
1 vs. 3	P-value	–	< 0.001	1.000	1.000	< 0.001
2 vs. 3	P-value	–	0.001	0.229	1.000	< 0.001

proportion of diseased cases that missed diagnosis by using native T1 cut-off. The results showed that the diseases that missed diagnosis by native T1 can be diagnosed by the cut-off of ECV data using actual Hct in 0% of amyloidosis, 28.8% of DCM, 12.2% of HCM, 25.0% of myocarditis, and 34.0% of CAD. If we used cut-off of ECV data using synthetic Hct, the diseases that missed diagnosis by native T1 can be diagnosed in 0% of amyloidosis, 53.6% of DCM, 25.0% of HCM, 10.0% of myocarditis, and 41.9% of CAD.

Sensitivity analysis was performed for the assessment of diagnostic yield results that might be influenced by the low prevalence of disease by propensity score matching of case and control at 1:1 ratio. Cases and controls were matched for age, gender, clinical presentation, and cardiovascular risk factors. The results of native T1 and ECV are shown in Additional files 1 and 2. The PPV increased whereas the NPV decreased.

#### Correlation and agreement for native T1 and ECV compared between different measurement approaches

The average native T1 compared between different measurement approaches (ROI drawn in mid LV myocardium and septum) showed a statistically significant correlation (R = 0.924, *p* < 0.001). The calculated ECV compared between mid LV and septum native T1 when using actual Hct showed a statistically significant correlation (R = 0.944, *p* < 0.001), and significant correlation was also found for ECV by synthetic Hct (R = 0.944, *p* < 0.001). The calculated mid LV ECV by actual Hct and synthetic Hct showed statistically significant correlation (R = 0.837, *p* < 0.001), and a significant correlation of both approaches was also found for septum ECV (R = 0.847, *p* < 0.001).

**Fig. 6:**
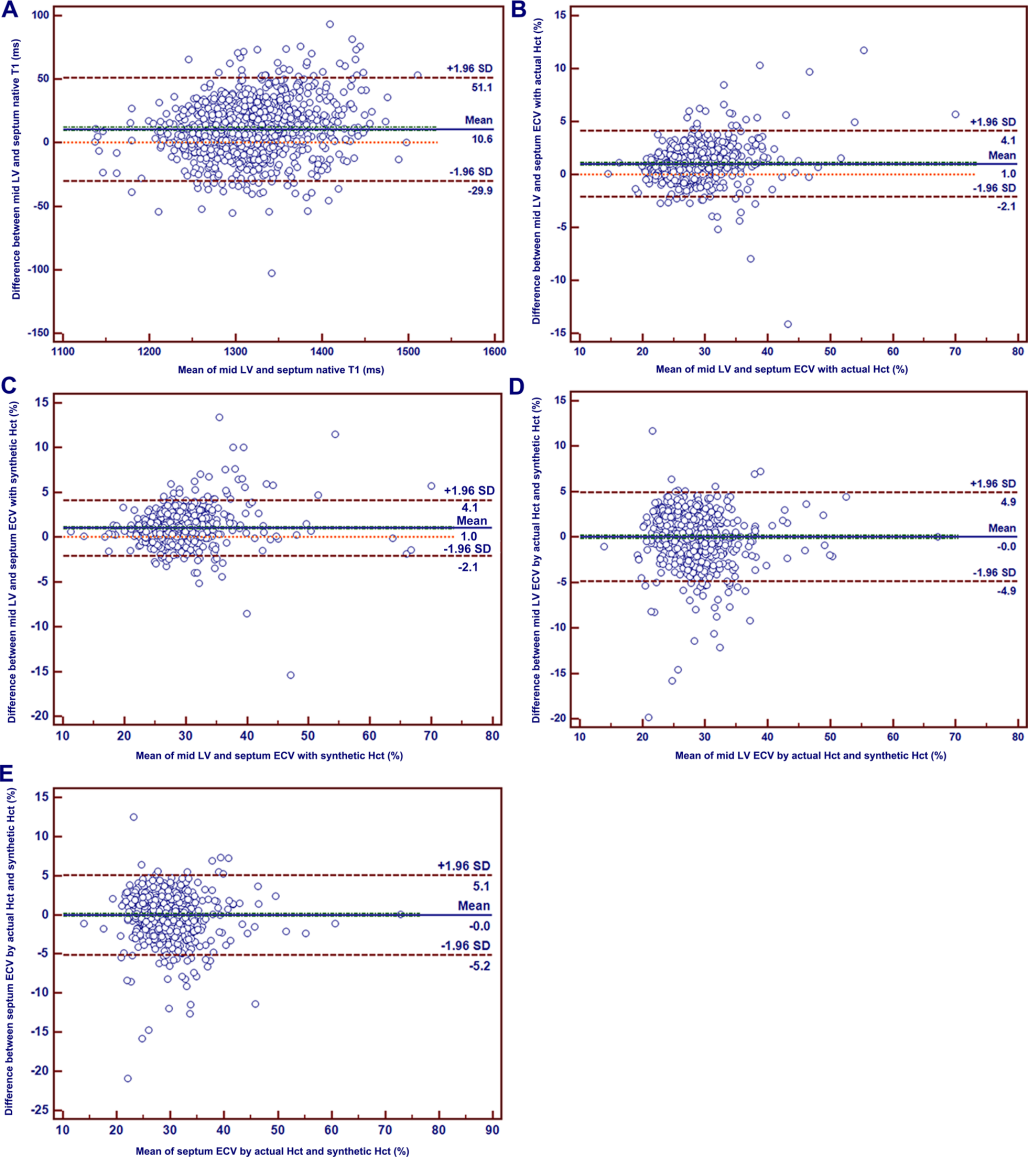
Bland-Altman comparison of different methods for measuring native T1 and ECV. **A** mid LV and septum native T1, **B** mid LV and septum ECV with actual Hct, **C** mid LV and septum ECV with synthetic Hct, **D** mid LV ECV by actual Hct and synthetic Hct, **E** septum ECV by actual Hct and synthetic Hct

The Bland-Altman plots confirmed the good agreement between septal and mid-LV T1 measurements and between ECV derived from the actual and synthetic Hct (Fig. 6).

## Discussion

Native T1 of myocardium was significantly higher in the myocardial disease and CAD groups compared to the control group. This study showed that myocardial disease and CAD can be differentiated from control group by means of native T1 mapping with good diagnostic accuracy. Amyloidosis had the highest AUC, sensitivity and specificity.

For differentiation between amyloidosis and controls the native T1 at septum and mid LV had a high diagnostic accuracy but the PPV of native T1 at mid LV was relatively low which may be related to the small number of disease subgroups. Karamitsos, et al. reported elevations in native T1 among patients with light chain (AL) cardiac amyloidosis that had definite cardiac involvement by endomyocardial biopsy, and in AL cardiac amyloidosis patients with uncertain or absent cardiac involvement by echocardiogram. Native T1 CMR was observed to be more sensitive than other methods for detecting cardiac amyloidosis [26]. The diagnosis of cardiac amyloidosis by using LGE had many limitations. First, LGE in patients with cardiac amyloidosis can have many patterns. It can be atypical or patchy especially in patients with early disease [27]. Second, amyloid deposition in the interstitium may reduce the contrast between blood and myocardium and the two components may null together. Thus, myocardium may appear normal in LGE images even the whole myocardium is diseased. The results of our study demonstrated that native T mapping and ECV are very accurate for the detection of cardiac amyloidosis. Native T1 mapping also have benefit in the detection of disease in case of impaired renal function that prohibit the use of CMR contrast agents and cannot acquire LGE images. Besides, T1 and ECV changes can provide earlier disease detection, even before LGE is detected [28].

In the present study, native T1 mapping was found to be an effective test for differentiating between controls and DCM with good diagnostic accuracy, specificity, and NPV, but not for sensitivity and PPV. The possible reason for limited sensitivity and PPV may be related to the accuracy of co-registration of thin-wall myocardium in DCM cases that is subjected to a potential measurement errors [29]. For differentiating HCM from controls, the average native T1 was found to have good diagnostic accuracy, specificity, and NPV, but it has limited sensitivity and PPV. Goebel, et al. reported significant difference in subgroup analysis of the average native T1 value between healthy heart patients and DCM patients (*p* < 0.001), and between healthy heart patients and HCM patients (*p* = 0.035). The AUC for the DCM group was 0.814 (*p* < 0.001), and the AUC for the HCM group was 0.688 (*p* = 0.067) [12] which is similar to the AUC results of our study.

Myocarditis patients usually have nonspecific signs, symptoms, and laboratory findings. Myocarditis is often diagnosed by exclusion of other cardiac causes. The diagnosis of myocarditis by CMR often requires multiple techniques, including early gadolinium enhancement, T2W, and LGE imaging. Updated recommendations of CMR criteria using novel CMR techniques, such as T1 mapping, T2 mapping, T2W image, and ECV, have more diagnostic accuracy [30]. The present study showed that native T1 mapping can differentiate myocarditis from controls with high accuracy, with an AUC of 0.710 (*p* = 0.003) for mid LV native T1, and with an AUC of 0.752 (*p* < 0.001) for septum native T1. Both ROI approaches showed high specificity and NPV, but limited sensitivity and PPV. However, we were not able to determine if native T1 mapping could distinguish between acute and convalescent myocarditis due to the small sample size in the acute myocarditis group (n = 2). Hinojar, et al., reported native T1 to be an independent discriminator between healthy patients and myocarditis patients, as well as for differentiating between acute and convalescent stage of myocarditis with high diagnostic accuracy, PPV, and NPV. The mean native T1 value was found to be higher in both the acute and convalescent myocarditis groups than in the healthy patient group [31]. The difference in results of Hinojar et al. and our study may be due to the smaller sample size of myocarditis in our study and may be related to the influence of the amount and location of LGE in myocarditis cases on T1 and ECV measurement.

In the CAD group, native T1 mapping test showed statistical significance in remote myocardium (excluding scarred segment) for mid LV short-axis approach and septal approach. The present study’s CMR imaging analysis protocol for the CAD group excluded subdivision segment with scar for native T1 analysis since the scar area since the scar would markedly increase native T1. However, lipomatous changes within the scar may decrease native T1 [32]. Previous studies showed inconsistent results on native T1 in remote myocardium of CAD cases. Some studies showed that native T1 of remote myocardium are similar to controls [12, 33, 34] but some studies showed an increased native T1 in remote myocardium [13]. Some patients might have a diffuse disease without a dense scar in the remote area and some may have ischemic myocardium which also can increase native T1 values [35].

Native T1 myocardium has acquisition without gadolinium-based contrast agent, which is an important advantage for patients with significant renal impairment who may be at increased risk for nephrogenic systemic sclerosis. The calculated ECV from T1 mapping reduces the variability of test results because of calculation from the ratio of change in myocardial T1 relative to blood pool T1 pre- and post-contrast [36]. The advantage of ECV is that it reduces systematic errors in technique, and results in less variability at different field strengths and across different vendor platforms [37]. The higher mean mid LV ECV value in the amyloidosis, DCM, HCM, and CAD groups demonstrated statistically significant difference from controls, similar to average native T1, except the same significant ability to differentiate from controls was not observed in the myocarditis subgroup. The mean ECV of myocarditis showed statistical significance when ECV was calculated using synthetic Hct. The observed differences in statistical significance relative to ECV between actual Hct and synthetic Hct in this study can be explained by the small number of patients and the lower number of patients in the actual Hct group.

The value of calculated ECV using actual Hct and synthetic Hct in each group showed concordant results with high degree of agreement which supported the report of the synthetic Hct formula by Fent, et al., [7].

### Study limitations

This study has some mentionable limitations. Firstly, no healthy volunteer data were included in this study. Patients having clinical indication for CMR without evidence of ischemia or infarction was used as the control group. It has been shown that patients with suspected CAD and negative adenosine CMR had an excellent prognosis with no major adverse cardiac event during follow up [38]. Results of meta-analysis also demonstrated a very excellent prognosis in patients with negative stress CMR study [39, 40]. Besides, in order to acquire ECV, post contrast T1 is needed for calculation. The injection of contrast agent in healthy volunteers is not practical in a clinical setup and it is difficult to obtain the ethical approval. A previous study that reported reference values of T1 in ‘normal healthy myocardium’ also used patients who were referred for CMR with negative CMR finding to define the reference value of T1 in normal [41]. Another study also used patients who were referred for CMR with normal CMR findings as control and compared to cardiovascular diseases [13]. They also mentioned that patients with negative CMR findings had T1 values similar to data of healthy volunteer from previous report [13]. The study objectives were designed to seek for the difference of T1 and ECV values among various groups of patients with myocardial disease and CAD in clinical practice rather than to distinguish T1 and ECV values between healthy volunteers and patients. The results of our study may be a more practical approach in real-world practice. Second, due to the retrospective study design, we used actual Hct within 6 months prior to CMR date to calculate ECV. Although we realized that certain factor such as hydration status could have effect on T1 relaxation time [42], we considered a 6-month period to be an acceptable duration. Furthermore, to reduce this limitation, we reported the calculated ECV with both actual Hct values and synthetic Hct values which were derived from T1 measurement of blood pool in the same sequence that myocardial T1 values were acquired. The Bland-Altman plot confirmed good agreement between the two methods, which supports that synthetic Hct-derived ECV may be used in place of conventional ECV in the cases that have missing Hct data. Third, native T1 mapping was performed in one mid LV short-axis slice, not the entire myocardial region. However, mid LV slice is recommended by standard guideline [6] and it should reflect pathology in patients with diffuse disease. Fourth, the number of patients in some subgroups of myocardial disease were rather small, so we could not test for differentiation between acute and convalescent myocarditis. Fifth, although we performed routine T1 mapping in the study population, we used the conventional criteria for the diagnosis of disease. Sixth, among patients in CAD group, this study aimed to measure the average native T1 and ECV of remote myocardium of CAD, not for infarcted area. Lastly, T1 mapping and ECV is well known to be sequence and technique dependent. This may limit the generalizability of the methodology. However, myocardial T1 mapping is a powerful clinical tool for soft tissue characteristic classification in spite of the absence of established reference values.

There were some strengths of this study. First, we reported data on a large number of patients. Second, we are the one of the few studies [24] that reported results from 3.0 Tesla CMR. Third, we compared results of myocardial disease and controls, and CAD versus controls. Fourth, we studied T1 mapping from ROI of mid LV slice and mid LV septum, and we used ECV from actual Hct and synthetic Hct.

## Conclusions

Although native T1 and ECV of patients with cardiomyopathy and CAD were significantly higher than controls, the values overlapped. The greatest clinical utilization was found for the amyloidosis group.

## Supplementary Information

**Additional file 1**. Cutoff values from receiver operating characteristic (ROC) curve analysis of native T1 in myocardial disease and CAD Case: control (1:1).

**Additional file 2**. Cutoff values from receiver operating characteristic (ROC) curve analysis of mid LV short-axis ECV in myocardial disease and CAD (1:1).

## Data Availability

The dataset that was used to support the results and conclusion of this study are included within the manuscript. Additional data are available upon contacting Rungroj Krittayaphong at rungroj.kri@mahidol.ac.th with the reasonable request.

## Abbreviations

CAD: Coronary artery disease
CMR: Cardiac magnetic resonance
DCM: Dilated cardiomyopathy
ECV: Extracellular volume fraction
HCM: Hypertrophic cardiomyopathy
Hct: Hematocrit
LGE: Late gadolinium enhancement
LV: Left ventricle
NPV: Negative predictive value
PPV: Positive predictive value
ROC: Receiver operating characteristics
ROI: Region of interest
