# Supplementary material for: Native T1 mapping and extracellular volume fraction for differentiation of myocardial diseases from normal CMR controls in routine clinical practice
Source: BMC Cardiovasc Disord. 2021 Jun 3;21:270. doi: 10.1186/s12872-021-02086-3 (PMC8173747; doi:10.1186/s12872-021-02086-3)
Supplement: Supplementary file 1 — Additional file 1. Cutoff values from receiver operating characteristic (ROC) curve analysis of native T1 in myocardial disease and CAD Case: control (1:1). [file 12872_2021_2086_MOESM1_ESM.pdf]

Additional file 1. Cutoff values from receiver operating characteristic (ROC) curve analysis of native T1 in myocardial disease and CAD Case: control (1:1)

	Cutoff (ms)	Sensitivity (%) (95% CI)	Specificity (%) (95% CI)	PPV (%) (95% CI)	NPV (%) (95% CI)
**Amyloidosis (n=11)**					
- Mid LV SAX native T1	1,340.5	100 (74.1-100.0)	72.7 (43.4-90.3)	78.6 (52.4-92.4)	100.0 (67.6-100.0)
- Septum native T1	1,370.3	81.8 (52.3-94.9)	90.9 (62.3-98.4)	90.0 (59.6-98.2)	83.3 (55.2-95.3)
**DCM (n=137)**					
- Mid LV SAX native T1	1,337.5	56.2 (47.8-64.2)	86.9 (80.2-91.5)	81.1 (72.0-87.7)	66.5 (59.3-73.0)
- Septum native T1	1,341.3	55.5 (47.1-63.5)	82.5 (75.3-87.9)	76.0 (66.8-83.3)	64.9 (57.6-71.6)
**HCM (n=112)**					
- Mid LV SAX native T1	1,332.5	53.6 (44.4-62.5)	86.6 (79.1-91.7)	80.0 (69.6-87.5)	65.1 (57.2-72.3)
- Septum native T1	1,346.3	53.6 (44.4-62.5)	88.4 (81.1-93.1)	82.2 (71.9-89.3)	65.6 (57.7-72.7)
**Myocarditis (n=17)**					
- Mid LV SAX native T1	1,354.5	41.2 (21.6-64.0)	94.1 (73.0-99.0)	87.5 (52.9-97.8)	61.5 (42.5-77.6)
- Septum native T1	1,359.8	52.9 (31.0-73.8)	94.1 (73.0-99.0)	90.0 (59.6-98.2)	66.7 (46.7-82.0)
**CAD**					
- Mid LV SAX native T1 (n=287)	1,341.5	30.7 (25.6-36.2)	93.4 (90.0-95.8)	82.2 (73.9-88.3)	57.7 (53.1-62.0)
- Septum native T1 (n=242)	1,342.8	36.8 (31.0-43.0)	81.7 (76.9-85.7)	62.7 (54.5-70.2)	60.8 (55.8-65.5)

A p-value<0.05 indicates statistical significance

Abbreviations: CAD, coronary heart disease; AUC, area under the ROC curve; CI, confidence interval; PPV, positive predictive value; NPV, negative predictive value; DCM, dilated cardiomyopathy; HCM, hypertrophic cardiomyopathy
