# Supplementary material for: Native T1 mapping and extracellular volume fraction for differentiation of myocardial diseases from normal CMR controls in routine clinical practice
Source: BMC Cardiovasc Disord. 2021 Jun 3;21:270. doi: 10.1186/s12872-021-02086-3 (PMC8173747; doi:10.1186/s12872-021-02086-3)
Supplement: Supplementary file 2 — Additional file 2. Cutoff values from receiver operating characteristic (ROC) curve analysis of mid LV short-axis ECV in myocardial disease and CAD (1:1). [file 12872_2021_2086_MOESM2_ESM.pdf]

Additional file 2. Cutoff values from receiver operating characteristic (ROC) curve analysis of mid LV short-axis ECV in myocardial disease and CAD (1:1)

	Cutoff (%)	Sensitivity (%) (95% CI)	Specificity (%) (95% CI)	PPV (%) (95% CI)	NPV (%) (95% CI)
**Amyloidosis**					
- Mid LV SAX actual ECV (n=8)	40.0	87.5 (52.9-97.8)	100.0 (67.6-100.0)	100.0 (64.6-100.0)	88.9 (56.5-98.0)
- Mid LV SAX synthetic ECV (n=11)	32.6	81.8 (52.3-94.9)	100.0 (74.1-100.0)	100.0 (70.1-100.0)	84.6 (57.8-95.7)
**DCM**					
- Mid LV SAX actual ECV (n=106)	28.8	51.9 (42.5-61.2)	79.2 (70.6-85.9)	71.4 (60.5-80.3)	62.2 (53.8-70.0)
- Mid LV SAX synthetic ECV (n=137)	27.0	71.5 (63.5-78.4)	56.9 (48.6-64.9)	62.4 (54.6-69.6)	66.7 (57.7-74.6)
**HCM**					
- Mid LV SAX actual ECV (n=84)	31.8	25.0 (17.0-35.2)	92.4 (84.4-96.5)	77.8 (59.2-89.4)	53.7 (45.3-61.8)
- Mid LV SAX synthetic ECV (n=112)	29.7	46.4 (37.5-55.6)	82.1 (74.0-88.1)	72.2 (61.0-81.2)	60.5 (52.6-67.9)
**Myocarditis**					
- Mid LV SAX actual ECV (n=13)	30.5	53.8 (29.1-76.8)	85.7 (60.1-96.0)	77.8 (45.3-93.7)	66.7 (43.7-83.7)
- Mid LV SAX synthetic ECV (n=17)	31.3	41.2 (21.6-64.0)	100.0 (81.6-100.0)	100.0 (64.6-100.0)	63.0 (44.2-78.5)
**CAD**					
- Mid LV SAX actual ECV (n=218)	27.9	48.2 (41.6-54.8)	60.7 (54.2-66.9)	54.4 (47.4-61.3)	54.6 (48.4-60.7)
- Mid LV SAX synthetic ECV (n=287)	27.3	54.0 (48.2-59.7)	55.9 (50.1-61.5)	54.8 (48.9-60.5)	55.1 (49.4-60.7)

A *p*-value<0.05 indicates statistical significance

Abbreviations: CAD, coronary heart disease; AUC, area under the ROC curve; CI, confidence interval; PPV, positive predictive value; NPV, negative predictive value; ECV, extracellular volume fraction; DCM, dilated cardiomyopathy; HCM, hypertrophic cardiomyopathy
